# Systems analysis reveals a transcriptional reversal of the mesenchymal phenotype induced by SNAIL-inhibitor GN-25

**DOI:** 10.1186/1752-0509-7-85

**Published:** 2013-09-03

**Authors:** Asfar S Azmi, Aliccia Bollig-Fischer, Bin Bao, Bum-Joon Park, Sun-Hye Lee, Gyu Yong-Song, Gregory Dyson, Chandan K Reddy, Fazlul H Sarkar, Ramzi M Mohammad

**Affiliations:** 1 Department of Pathology, Karmanos Cancer Institute, Wayne State University School of Medicine, John R, HWCRC 732, Detroit, MI, 4100, USA; 2 Department of Oncology, Karmanos Cancer Institute, Wayne State University School of Medicine, Detroit, MI, USA; 3 Department of Molecular Biology, College of Natural Science, Pusan National University, Busan, Korea; 4 College of pharmacy, Chungnam National University, Daejeon, Korea; 5 Department of Computer Sciences, Wayne State University, Detroit, MI, USA; 6 Hamad Medical Corporation, Doha, Qatar

**Keywords:** SNAIL, EMT, MET, Snail inhibitor, Systems biology, Network analysis, Pathway analysis

## Abstract

**Background:**

HMLEs (HMLE-SNAIL and Kras-HMLE, Kras-HMLE-SNAIL pairs) serve as excellent model system to interrogate the effect of SNAIL targeted agents that reverse epithelial-to-mesenchymal transition (EMT). We had earlier developed a SNAIL-p53 interaction inhibitor (GN-25) that was shown to suppress SNAIL function. In this report, using systems biology and pathway network analysis, we show that GN-25 could cause reversal of EMT leading to mesenchymal-to-epithelial transition (MET) in a well-recognized HMLE-SNAIL and Kras-HMLE-SNAIL models.

**Results:**

GN-25 induced MET was found to be consistent with growth inhibition, suppression of spheroid forming capacity and induction of apoptosis. Pathway network analysis of mRNA expression using microarrays from GN-25 treated Kras-HMLE-SNAIL cells showed an orchestrated global re-organization of EMT network genes. The expression signatures were validated at the protein level (down-regulation of mesenchymal markers such as TWIST1 and TWIST2 that was concurrent with up-regulation of epithelial marker E-Cadherin), and RNAi studies validated SNAIL dependent mechanism of action of the drug. Most importantly, GN-25 modulated many major transcription factors (TFs) such as inhibition of oncogenic TFs Myc, TBX2, NR3C1 and led to enhancement in the expression of tumor suppressor TFs such as SMAD7, DD1T3, CEBPA, HOXA5, TFEB, IRF1, IRF7 and XBP1, resulting in MET as well as cell death.

**Conclusions:**

Our systems and network investigations provide convincing pre-clinical evidence in support of the clinical application of GN-25 for the reversal of EMT and thereby reducing cancer cell aggressiveness.

## Background

The epithelial-to-mesenchymal transition (EMT) and the reverse process, termed the mesenchymal-to-epithelial transition (MET), plays a central role in cancer progression and cell death [1]. Cells undergoing EMT are characterized by their elongated morphology, inherent aggressiveness, propensity to maintain in long term cell culture conditions that is reminiscent of stem cell characteristics, which is also associated with resistance to standard chemotherapies and targeted therapies [2]. Regimens designed to hit bulk of the tumor cells, in most cases do not eliminate these EMT sub-population of cells and this has been suggested to be the underlying cause for drug resistance and tumor recurrence [3]. Therefore, targeted elimination of these EMT cells would be an important prerequisite for achieving optimal results for successful anti-cancer therapy.

SNAIL family of proteins have been shown to play an important role in the acquisition of malignant (aggressiveness) phenotype of epithelial tumors [4]. SNAIL homologues are thought to act as transcriptional repressors and show a conserved function in mesoderm development from flies to mammals [5]. Their role in de-lamination and migration is mediated by triggering the processes that leads to the acquisition of EMT by directly repressing the transcription of E-cadherin [6]. Activation of SNAIL has been shown in pathological specimens at the invasive front of chemically induced mouse skin tumors, mammary, ovarian and in human breast carcinomas [7]. A number of different signaling pathways such as TGF-β, BMP, FGF and Wnt signaling have been implicated in the induction of Snail family members during the process of EMT [8]. Based on its critical role, targeted inhibition of SNAIL proteins has been investigated in pre-clinical setting as a therapeutics strategy to reverse EMT phenotype [9].

Even though many different cell models have been developed that mimic the genotypic and phenotypic characteristics of EMT; however, the Weinberg’s HMLE-SNAIL models stand out to be very useful among many others [10]. This is in part due to the fact that EMT induction in HMLEs is driven by over-expression of a single mesenchymal driver i.e. SNAIL [10]. These and related HMLE cells have been well characterized for their unique expression signatures that promote EMT in earlier studies. These cells have also been shown to form spheroids, maintain survival in long term cell culture condition, and carry markers of cancer stem-like cells (over-expression of Vimentin, ZEB1, TWIST 1 and TWIST 2 and down-regulation of epithelial markers E-Cadherin) [10]. Therefore, HMLEs could serve as an excellent tool for investigating perturbations induced by agents specific towards SNAIL (SNAIL inhibitors).

Previously, our group has developed a specific SNAIL-p53 interaction inhibitor GN-25 [11], and this drug was originally designed to disrupt SNAIL-p53 interaction, thereby removing the p53-post-translational regulatory control and rescuing the cell surveillance functions of this master regulator [12]. GN-25 is cancer cell specific and does not induce growth inhibition or apoptosis in normal immortalized cells. In the current study, we found that GN-25 could reverse the EMT phenotype and could also induce apoptosis in cancer cells, which prompted us to further investigate the mechanism of action of GN-25 against HMLE-SNAIL model. In this report, we performed network analysis using the HMLE-SNAIL models (EMT phenotypic cells induced by stable transfection with SNAIL) pre- and post-treatment with a SNAIL inhibitor GN-25. Our network analysis and biological validation showed that (a) EMT in HMLE-SNAIL arises through a complex crosstalk between different mesenchymal phenotype promoting networks of pathways, and (b) SNAIL inhibitor induces a coordinated set of perturbations that re-align the EMT networks with reversal to MET phenotype.

## Results

### GN-25 induces growth inhibition in SNAIL-transduced HMLE cell line models

**Figure 1:**
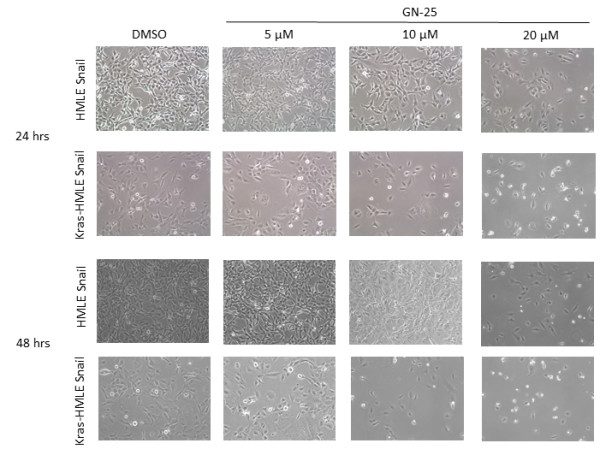
**GN-25 reverses EMT phenotype.** HMLE-SNAIL, Kras-HMLE and Kras-HMLE-SNAIL cells were exposed to different concentrations of GN-25 as indicated and incubated for 24 and 48 hrs. Photomicrographs were taken using light microscope at different time points. Note: GN-25 resulted in growth inhibition as well as caused reversal of EMT phenotype to epithelial phenotype.

**Figure 2:**
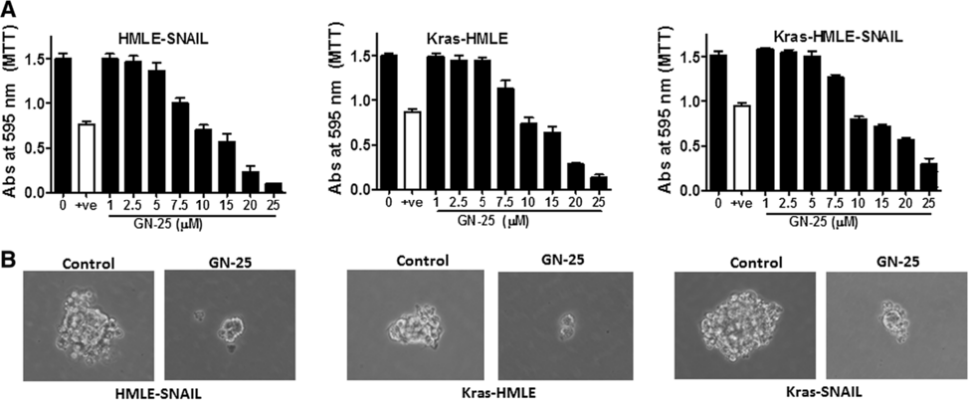
**GN-25 induces growth inhibition and suppresses spheroid formation in SNAIL-transduced HMLE models. (A)** HMLE-SNAIL, Kras-HMLE and Kras-HMLE-SNAIL cells were exposed to different concentrations of GN-25 or Quercetin as + ve control as indicated and incubated for 72 hrs. Growth inhibition was evaluated using MTT assay. **(B)** Cells were allowed to grow as spheroids as described under Methods section. Once established, the spheroids were exposed to GN-25 (20 μM). Note: GN-25 suppresses growth and sphere-forming capacity of Snail-HMLEs. Figures are representative of three independent experiments.

Structure of GN-25 has been published previously [13]. We performed a time course evaluation of GN-25 induced morphological changes in HMLE cell pairs. Figure 1 shows that compared to control cells (mesenchymal and elongated, typical feature of EMT phenotypic cells), GN-25 showed reversal from EMT to Mesenchymal-to-Epithelial Transition (MET) phenotype in the SNAIL and Kras plus SNAIL transduced HMLE cell lines (epithelial round cells in higher dose treatments at 24 and 48 hrs). Next, we investigated GN-25-induced growth inhibition by MTT assay. As can be seen from the results presented in Figure 2A, all three cell lines (HMLE-SNAIL, Kras-HMLE and Kras-HMLE-SNAIL) showed a statistically significant growth inhibition by GN-25 treatment. Quercetin a known indirect and weak inhibitor of SNAIL was used as a positive control and showed growth inhibitory activity at 20 μM concentration.

### GN-25 suppresses spheroid formation in SNAIL transduced HMLE cells

We then investigated whether our drug could suppress the propensity of SNAIL-Transduced Kras-HMLEs to form spheroids using sphere-forming assay. As can be seen from of photomicrographs presented in Figure 2B (control, DMSO-treated spheres), all cells were able to grow as spheroids in 3D culture. However, upon 20 μM GN-25 treatment there was a marked disintegration of spheres in all three cell lines tested (Figure 2B left panels). These results clearly showed that SNAIL inhibitor GN-25 not only suppresses growth of HMLEs cells, but also reduces their ability to form spheroids in 3 D culture.

### GN-25 induces apoptosis in HMLE EMT cell models

**Figure 3:**
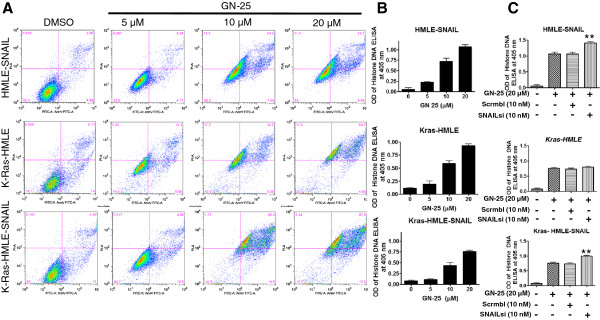
**GN-25 induces apoptosis in HMLE cells.** HMLE cells were exposed to different concentrations of GN-25 as indicated and incubated for 72 hrs. Apoptosis was evaluated using Annexin V FITC assay **(A)** and Histone DNA ELISA assays **(B)**. Values are representative of three independent experiments. **(C)** Cells were incubated with either scrambled siRNA or SNAI2 siRNA (Santa Cruz Biotechnology) for 24 hrs according to previously published methods [26], siRNA silenced cells were seeded at a density of 50,000 cells per well in six well plate and further exposed to GN-25 (20 μM) for additional 72 hrs. Apoptosis was evaluated using Histone DNA ELISA assay. Graphs are representative of three independent experiments. ** represents statistically significant p < 0.01 when compared to GN-25 alone treatment.

Once the MET inducing, growth inhibitory and spheroid suppressive activity of GN-25 against SNAIL transduced HMLE cell line models were confirmed, we evaluated whether the drug could induce apoptosis in these cells using Annexin V FITC and Histone DNA ELISA assay. As can be seen from the results presented in Figure 3A, we observed a progressive increase in apoptotic cell death with increasing concentrations of GN-25 (treated for 72 hrs) in HMLE-SNAIL (25.1% early stage apoptosis), Kras-HMLE (63.5% early stage apoptosis) and Kras-HMLE-SNAIL (67.8% early stage apoptosis) cells. Similar results were obtained using the Histone DNA ELISA assay with the three cells showing a statistically significant increase in the induction of apoptosis with increasing doses of GN-25 (Figure 3B). These results suggest that GN-25 not only reverses EMT phenotype but this is also accompanied by the induction of apoptosis in the HMLE cells.

### Effect of SNAI2 siRNA on GN-25 activity

As SNAIL is the primary target of GN-25, we investigated how the GN-25 drug compared with the effects of SNAIL-targeted siRNA on the induction of apoptosis. As can be seen from the results presented in Figure 3C, the apoptotic potential of GN-25 was similar to that of control siRNA. However, introduction of SNAI2-targeted siRNA when combined with GN-25 appears to enhance the degree of apoptosis. In Kras-HMLE cells (that have not been transduced with SNAIL), the siRNA treatment did not enhance the activity of the drug. These results provided the proof-of-concept showing that SNAIL protein is a direct target of GN-25. However, further in-depth analysis is needed to analyze what apparently constitute the broader effects of GN-25 treatment in order to understand the genes and functions that are deregulated during the reversal of EMT to MET after treatment with GN-25, and for which microarray and pathway modeling experiments were performed.

### Systems-level analyses of gene expression level changes induced by GN-25 treatment specifically in SNAIL-transduced HMLE cell lines

**Figure 4:**
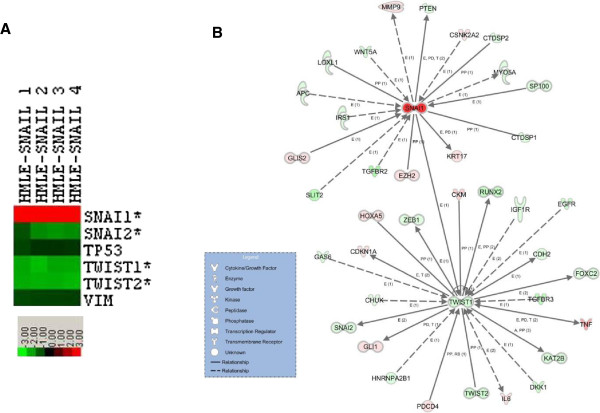
**The expression of EMT factors is changed by GN-25 treatment in Kras-HMLE-SNAIL cells. (A)** Six EMT-associated genes were identified among genes that showed a significant LogRatio detection p-value in an Agilent two-color gene expression microarray (p ≤ .001). The heat map of Log2 gene expression ratios shows how many EMT-associated genes were up-regulated (red) or down-regulated (green) by treatment Kras-HMLE-SNAIL cells with GN-25 across four biological replicates. Genes with an asterisk (*) were among those shown to be uniquely regulated by GN-25 treatment in Kras-HMLE-SNAIL cells as determined by the ANOVA analysis that compared response to GN-25 treatment versus vehicle control in Kras-HMLE-SNAIL and Kras-HMLE cells (p ≤ .001, Benjamin-Hochberg FDR multiple test correction p ≤ .05). **(B)** The functional relationships for these genes and a substantial number of others linked to them from the Kras-HMLE-SNAIL-specific data set are mapped in the interaction network where the edges between genes represent data and peer-reviewed results contained in the Ingenuity Systems’ curated knowledgebase data set.

Microarray experiments and analyses (described in detail under Methods), were carried out to identify significant gene expression level changes that were uniquely affected by GN-25 treatment in the context of the transformed SNAIL over-expressing cell line, Kras-HMLE-SNAIL, compared to GN-25 effects in the control Kras-HMLE cell line. The net result showed a set of 2,737 genes (functionally annotated gene transcripts) with corresponding Kras-HMLE-SNAIL expression ratios (GN-25-treated versus vehicle control) (Additional file 1: Figure S1). The genes were examined in the follow-up systems-level analyses using Ingenuity Systems® software (Redwood, CA) to investigate the predicted on-target impact of GN-25 treatment and to infer additional downstream targets and broader biological consequences of GN-25 treatment in the SNAIL transduced cells. A global gene ontology analysis using Ingenuity Systems® software predicted that GN-25 treatment showed decreased activation state for a significant functional annotations including formation of cellular protrusions, transformation, migration of tumor cell lines and cell movement (Additional file 2: Table S1), all are consistent with the conclusion that GN-25 reverses the EMT or rather induces MET phenotypic and genotypic changes. Gene expression data were queried to see if GN-25 treatment impacted known EMT-associated factors in Kras-HMLE-SNAIL cells specifically. Of eleven known EMT-associated genes compiled from the literature, six were identified among the genes that were significantly up-regulated or down-regulated by treatment with GN-25 in Kras-HMLE-SNAIL cells—they are SNAIL family members SNAI1 and SNAI2, and TWIST family members TWIST1 and TWIST2 (Figure 4A). The down-regulation of SNAI2, TWIST1 and TWIST2 is consistent with evidence that TP53 down-regulates TWIST expression and, in turn, down-regulates SNAI2 expression. We can only speculate that the up-regulation of SNAI1 may be the result of a feedback mechanism that up-regulates SNAI1 when signaling resulting from SNAIL/TP53 interaction is inhibited by GN-25. The regulatory relationships for these genes and a substantial number of others from the Kras-HMLE-SNAIL-specific data set that are functionally associated with them are mapped in the interaction network in Figure 4B where the edges between genes represent data and peer reviewed results contained in the Ingenuity Systems’ curated knowledgebase.

**Figure 5:**
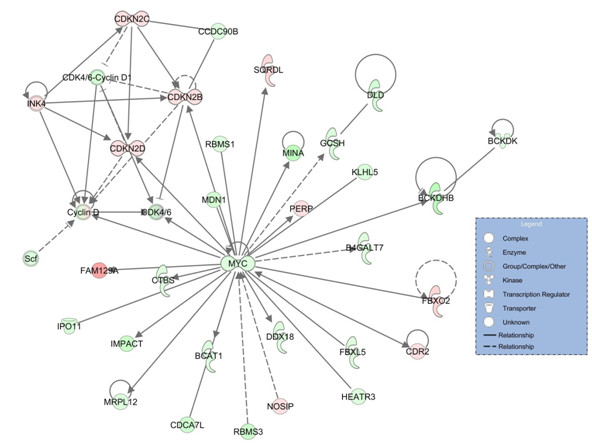
**Genes in the Kras-HMLE-SNAIL-specific data set linked to Myc function.** Genes that are differentially and significantly regulated by GN-25 treatment in Kras-HMLE-SNAIL cells specifically compared to Kras-HMLE cells that are associated with Myc transcription factor function. Perspective is fold-change (treated/untreated), red up-regulated, green down-regulated. From Ingenuity Systems® analysis.

In additional analysis we looked specifically at GN-25-induced expression level changes and the predicted activity state for transcription factors in Kras-HMLE-SNAIL-unique data set. The transcription factor activity prediction tool in the Ingenuity Systems® software uses an algorithm that weighs the direction of transcript level changes in the data set of interest and an empirical database to elucidate the genes that are likely regulated by a given transcription factor. A regulation z-score and statistical test are factored into a prediction for the activation state of a transcription factor—the score reflects the numbers of target gene candidates that are changed in the experiment data set and the number of those that are changed in a direction consistent with the Ingenuity Systems’ database. According to the results presented in Table 1, TP53 is predicted to be activated by GN-25 treatment, which is consistent with the on-target effect of GN-25 mediated disruption of the SNAIL/TP53 protein-protein interaction. Furthermore, TWIST1 is predicted to be inhibited, which correlates with the observed down-regulation of TWIST1 expression and the down-regulation of TWIST1 target SNAI2. Furthermore, the analysis revealed that GN-25 treatment of the Kras-HMLE-SNAIL transformed cells was very likely inhibiting MYC and glucocorticoid receptor (NR3C1) transcription factors and their related functional outcomes. Figure 5 displays the MYC-regulated gene expression network predicted to be inhibited by GN-25 treatment in Kras-HMLE-SNAIL cells. A greater understanding of possible cancer relevance for these discoveries came from subsequent analysis using Oncomine™ [14]. Uploading the list of the identified MYC-regulated genes from the analyzed data set into a survey of the Oncomine™ database revealed that the MYC-targeted genes in the data set significantly overlapped with gene sets associated with an exceptionally poor prognosis, including breast cancer clinical outcome i.e. dead at 3 years (Additional file 3: Table S2).

**Table 1:** Impact of GN-25 treatment on transcription factor programs in Kras-HMLE-SNAIL cells

**Transcription factor**	**Expression treated/untreated**	**Predicted activation state**	**Regulation z-score**	**p-value of overlap**
TBX2	-2.005	Inhibited	-2.978	1.91E-04
MYC	-2.245	Inhibited	-2.912	1.52E-08
NR3C1	-3.751	Inhibited	-2.654	1.21E-08
TWIST1	-4.096	Inhibited	-2.028	3.92E-02
GLI1	2.264	Activated	2.073	3.00E-02
SMAD7	2.005	Activated	2.079	4.17E-06
DDIT3	8.076	Activated	2.110	1.06E-02
CEBPA	4.142	Activated	2.189	9.86E-05
HOXA5	3.132	Activated	2.189	1.27E-01
TFEB	2.098	Activated	2.339	3.92E-02
IRF1	3.651	Activated	2.506	2.84E-02
XBP1	2.026	Activated	3.415	4.11E-01
IRF7	3.915	Activated	3.894	1.32E-04
TP53	NS	Activated	3.502	4.57E-18

Transcription factors whose expression levels (fold-change, treated versus untreated) and predicted activation state are significantly changed by GN-25 treatment in HMLE-SNAIL cells specifically compared to HMLE cells. Although the expression levels of TP53 was not significantly (NS) changed by GN-25 treatment in HMLE-SNAIL cells, its function was predicted to be inhibited in untreated HMLE-SNAIL cells, but activated by GN-25 treatment. Myc-target genes in the data set significantly overlap with a gene set with clinical outcome (dead at 3 years) (Additional file 3: Table S2). GN-target genes in the data set overlap significantly with the 3 year clinical outcome metastatic outcomes. Oncomine query matching VV and Koa, respectively.

### Biological validation of network genes at expression level

**Figure 6:**
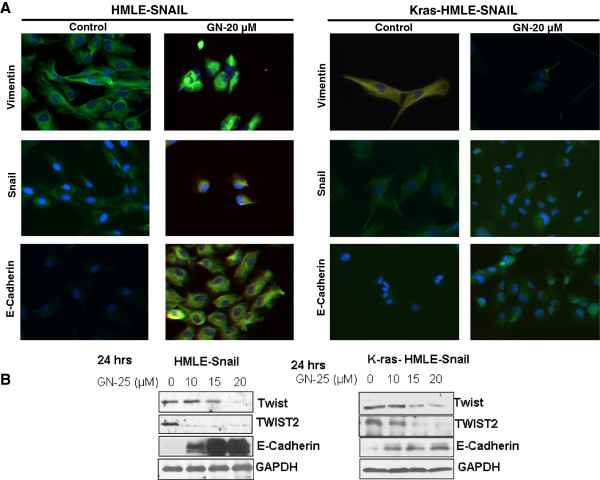
**Biological validation of expression datasets at the protein level. (A)** Confocal analysis of HMLE-SNAIL and Kras-HMLE-SNAIL cells post GN-25 treatment. Cell were exposed to indicated concentrations of GN-25 for 24 hrs in 4 well chambered slides and immunofluorescence assay was performed according to published methods . Slides were stained with either vimentin (cell signaling), Snail (Cell signaling) or E-Cadherin (cell signaling) antibodies overnight. Secondary Antibody staining was performed for 2 hrs using anti-mouse Alexafluor antibody (Invitrogen). **(B)** Western blot analysis of HMLE-SNAIL and Kras-HMLE-SNAIL cells exposed to indicated concentrations of GN-25 for 24 hrs. The blots were probed for TWIST1, TWIST2 and E-Cadherin. GAPDH was used as a loading control. Blots are representative of two independent experiments.

Once the GN-25 induced network signatures were obtained, we validated the expression of their constituents by confocal microscopy and western blot analysis. As can be seen from results in Figure 6A, GN-25 treatment resulted in condensed expression of Vimentin (on nuclear envelope) and suppression of SNAIL expression in HMLE-SNAIL and Kras-HMLE-SNAIL cells. Most importantly, suppression of EMT markers was concurrent with re-expression of E-Cadherin (Lower panels). In western blot experiments we observed suppression of EMT markers in both HMLE-SNAIL and Kras-HMLE-SNAIL cells Figure 6B. The major drivers of EMT, i.e. TWIST1 and TWIST2 were suppressed with progressive increasing concentrations of GN-25. On the other hand the expression levels of epithelial marker E-cadherin and EGFR were found to be up-regulated. The results clearly demonstrate that GN-25-induced growth inhibitory, MET and apoptosis outcomes coincide with reversal of the expression of EMT factors and re-expression of epithelial-associated factors.

## Discussion and conclusion

Here we report, for the first time, the network analysis and biological validation of the EMT reversing perturbations induced by a SNAIL inhibitor GN-25 in the HMLE-based model system. Using systems level investigations, we showed that GN-25 induces MET and consequently growth inhibition and induction of apoptosis in SNAIL-transduced HMLE cells through coordinated suppression of EMT network genes. Our findings also highlight the role of a number of secondary players that cumulatively support GN-25’s mechanism of action. These findings demonstrate that the successful design of drugs against EMT should not only be focused on EMT specific genes but additional secondary networks that may require a promiscuous targeting by drugs that have pleiotropic mode of action.

EMT confers mesenchymal properties to epithelial cells, and this has been closely associated with aggressiveness of carcinoma cells [15]. Emerging research shows that EMT programs are orchestrated not by one, but a set of pleiotropically acting transcription factors (TFs) [16]. The actions of these EMT-TFs enable the propensity for early steps of metastasis; local invasion and subsequent dissemination of carcinoma cells to distant sites. The genetic and epigenetic mechanisms that regulate the activation of EMT-TFs and the traits they induce are areas under intensive investigation. Such studies are expected to provide new opportunities for therapeutic intervention and may help to overcome tumor heterogeneity and therapeutic resistance.

The discovery and development of HMLE cell line models were facilitated through introduction of hTERT, which encodes the catalytic subunit of the human telomerase holoenzyme, as well the SV40 early region [10]. Additionally, introduction of EMT promoting genes such as snail and twist in these models facilitated the reprogramming of the transduced and transformed HMLE cells to give rise to EMT phenotype [10]. These developments have helped to molecularly, understand the basis of this complex phenomenon. This has, in turn, driven the research on pharmaceutical strategies that target reversal of EMT. Nevertheless, EMT is a complex process arising from the de-regulations in complex biological networks [17]. These EMT biological networks cannot be investigated in isolation (using reductionist approaches), and thus it requires advanced, holistic and systems level analyses. This is especially needed in order to develop drugs that reverse EMT.

While Weinberg’s EMT cell line models have been the subject of individual-set of differentially expressed (DE) gene analyses using the t-test and the F-test, these are still insufficient knowledge to interrogate the EMT phenomena, which is in part due to the presence of additional genes that do not meet the DE criteria. Such analysis cannot extract EMT-specific characterization of mesenchymal pathway genes; i.e. identifying the distinguishing set of mesenchymal patterns in the entire co-expressed gene groups that may be specific to EMT only. Additionally, to date there are no drug related studies reported that showed alterations in broader effects on EMT signaling or functional networks. Here, we showed a network-based differential analysis model for analyzing the topological differences between two gene networks constructed from the expression data from GN-25 treated cells. We selected Kras-HMLE-SNAIL over HMLE-SNAIL for our network analysis since earlier it was shown that Kras transforms HMLE cells and drives SNAIL expression through the activation of Gli [10]. Supporting this notion, Morel and colleagues have shown that sequential retroviral-mediated expression of the telomerase catalytic subunit (giving rise to HMEC/hTERT cells), SV40 large T and small t antigens (HMLE cells) and an oncogenic allele of H-Ras, H-Ras^V12^ (HMLER cells) accelerates EMT [18]. This allowed us to ask two questions: (1) what are the networks modulated in response to our SNAIL inhibitor, and (2) is there a compensatory influence of oncogenic Ras on GN-25 activity.

Our in-depth network analyses provided insights for multiple factors that are involved in what can be considered on-target drug predictions (e.g., the functional up-regulation of certain transcription factors resulted from GN-25 treatment) consistent with what is known about EMT. The analysis also presented broader secondary downstream or pleiotropic effects of the drug that may enhance its effectiveness in reversing EMT. Further, the analysis points to pathways where *de novo* or acquired resistance mechanisms may be the important route. These preliminary investigations demonstrate that drug design guided solely by presumed targets and differentially expressed genes may not be successful in reversing EMT due to the presence of multiple factors that function together to reinforce the phenotype. However, as shown by our network results, agents such as GN-25, with far-reaching effects (i.e. with inherent network pharmacology properties), can better serve the purpose in reversing EMT phenotype by not only directly targeting an assumed target and differentially expressed genes, but also secondary yet important signaling pathways or functional networks. In conclusion, our network investigations provided convincing pre-clinical rationale in support of the clinical application of GN-25 and related agents for the treatment of EMT cells in order to overcome therapeutics resistance of aggressive and metastatic cancers.

## Methods

### Cell lines and culture conditions, and research reagents

SNAIL-transduced HMLEs (HMLE-SNAIL, Kras-HMLE and Kras-HMLE-SNAIL) were generously provided by Dr. Robert Weinberg, Whitehead Institute, Massachusetts. SNAIL inhibitor GN-25 was developed as documented previously [11]. Quercetin; an indirect inhibitor of SNAIL was purchased from SIGMA (St Louis USA). Primary antibodies for SNAIL, Vimentin, TWIST1 and TWIST2 were purchased from Cell Signaling (Danvers, MA). All the secondary antibodies were obtained from Sigma (St. Louis, MO).

### Cell growth inhibition by 3-(4,5-dimethylthiazol-2-yl)-2,5-diphenyltetrazolium bromide assay (MTT)

SNAIL-transduced HMLE cells were seeded at a density of 5 × 10^3^ cells per well in 96-well micro-titer culture plates. After overnight incubation, medium was removed and replaced with fresh medium containing GN-25 at indicated concentrations (0–25 μM) diluted from a 10 mM stock or Quercetin (used as positive control at 20 μM). After 72 hours of incubation, MTT assay was performed by adding 20 μL of 3-(4,5-dimethylthiazol-2-yl)-2,5-diphenyltetrazolium bromide (MTT) Sigma (St. Louis, MO) solution (5 mg/mL in PBS) to each well and incubated further for 2 hours. Upon termination, the supernatant was aspirated and the MTT formazan formed by metabolically viable cells was dissolved in 100 μL of isopropanol. The plates were gently rocked for 30 minutes on a gyratory shaker, and absorbance was measured at 595 nm using a plate reader (TECAN, Durham, NC).

### Sphere formation/disintegration assay

Briefly, single-cell suspensions of HMLE-SNAIL, Kras-HMLE and K-ras-HMLE-SNAIL were plated on ultra–low adherent wells of 6-well plates (Corning) at 1,000 cells per well in sphere formation medium (1:1 DMEM/F12 medium supplemented with B-27 and N-2; Invitrogen). After 7 days, the spheres were collected by centrifugation (300 xg, 5 minutes) and counted. The proportion of sphere-generating cells was calculated by dividing the number of spheres by the number of cells seeded. Single-cell suspensions of spheres were plated at 500 cells per well in the sphere formation medium. After 1 or 3 weeks of incubation with GN-25, secondary spheres were harvested for counting as described above. For sphere disintegration assay, 1,000 cells per well on ultra–low adherent wells of 6-well plate were incubated for a total of 10 days following 5 days of drug treatment, and the cells were harvested as described previously [19]. The spheres were collected by centrifugation and counted under a microscope as described above.

### Quantification of apoptosis by histone DNA ELISA and annexin V FITC assay

Cell Apoptosis was detected using Annexin V FITC (Biovision Danvers MA) and Histone DNA ELISA Detection Kit (Roche, Life Sciences) according to the manufacturer's protocol. HMLE cells were seeded at a density of 50,000 cells per well in six-well plates in 5 ml of corresponding media. 24 hrs after seeding the cells were exposed to GN-25 at different concentrations for 72 hrs. At the end of treatment period cells were trypsinized and equal numbers were stained with Annexin V and Propidium Iodide. The stained cells were analyzed using a Becton Dickinson flow cytometer at the Karmanos Cancer Institute Flow cytometry core. The second apoptosis assay quantifies histone-complexed DNA fragments (mono- and oligonucleosomes) from the cytoplasm of cells after the induction of apoptosis or when released from necrotic cells. Since the assay does not require pre-labeling of cells, it can detect internucleosomal degradation of genomic DNA during apoptosis. All procedures were performed according to our previously published protocol [20].

### Immunofluorescence and confocal microscopy

Cells were grown on glass chamber slides and exposed to GN-25 at indicated concentrations for 24 hrs. In another set of experiments, at the end of the treatment the cells were fixed with 10% paraformaldehyde for 20 min. The fixed slides was blocked in TBST and probed with primary and secondary antibody according to our previously published methods [21]. The slides were dried and mounting medium was added to it and covered with a coverslip and were analyzed under an inverted fluorescent microscope. A total of three independent experiments were performed.

### Western blot analysis

HMLE-SNAIL and Kras-HMLE-SNAIL cells were grown in 100 mm petri-dishes over night to ~70% confluence. Next day, cells were exposed to indicated concentrations of GN-25 for 24 hrs followed by extraction of protein for western blot analysis. Preparation of cellular lysates, protein concentration determination and SDS-PAGE analysis was done as described previously [22].

### siRNA and transfection

To study the effect of SNAIL silencing on activity of GN-25, we utilized siRNA silencing technology. SNAI2 siRNA and control siRNA were obtained from Santa Cruz Biotechnology. Cells were transfected with either control siRNA or SNAIL siRNA for 24 hrs using Lipofectamine 2000 (Invitrogen) according to the manufacturer’s protocol. All procedures have been standardized and published previously [23]. After the siRNA treatment period, cells were further treated with GN-25 (at IC_50_ concentration) in 96-well plates for MTT and 6-well plates for Annexin V FITC assays, respectively. Knock-down efficiency was evaluated by western blot analysis.

### Microarrays and gene expression data analysis

K-ras-HMLE-SNAIL cells were plated so that they reached 75% confluence after 3 days. At this point, cultures were treated with or without GN-25 (15 μM). Total RNA was isolated from four sets of parallel plated culture plates, treated with or without GN-25, at 24 hours after the addition of inhibitor. Media was changed the day after plating and at the start of treatment. Total RNA quantity and quality was determined by analysis using the NanoDrop 1000 and Agilent Bioanalyzer (Agilent Technologies). All analyzed samples had RIN scores ≥ 7. Whole-genome expression levels were analyzed by a two-color microarray-based approach. A treated and untreated sample for one cell line was combined and hybridized to Agilent 4x44k human arrays and scanned with the Agilent G2505B microarray scanner system. Data quality was assessed, and data were processed by Agilent Feature Extraction software that produced expression data measures including LogRatio expression levels, LogRatio Error and P Value LogRatio. Features included in further analysis were annotated, gene-level that passed a p Value LogRatio cut-off ≤ 0.001. ANOVA analysis and multi-test correction (Benjamin-Hochberg p ≤ 0.05) was done using Partek software to compare the 2 sets of 4 two-color arrayed replicates (the 2 sets were Kras-HMLE and Kras-HMLE-SNAIL cell lines), to identify gene expression level changes (≥2 fold-change) that were uniquely affected by GN-25 treatment in the context of SNAIL-transduced Kras-HMLE cell line compared to GN-25 effects on the expression in the Kras-HMLE cell line (ANOVA p ≤ 0.001). The net result was a set at 2,737 genes, and corresponding HMLE-SNAIL expression ratios (GN-25-treated versus vehicle control) that were examined in follow-up systems-level analyses.

### Availability of supporting data

The supporting microarray data is publicly available and can be freely accessed at the following link: http://www.ncbi.nlm.nih.gov/geo/query/acc.cgi?acc=GSE44397.

## Competing interests

The authors declare no competing interests.

## Authors’ contributions

AAS conceived hypothesis, designed experiments and performed MTT assay, apoptosis assay, siRNA experiments, immunofluorescence experiments and western blot analysis; BFA performed microarray analysis, pathway analysis and Oncomine analysis; BB performed spheroid formation assays; PBJ, LSH and SYJ developed and synthesized the drug GN-25; DG performed statistical evaluation, read and edited the paper; RC provided intellectual input, read and edited paper; SFH conceived hypothesis, designed experiments, analyzed data, read and edited the manuscript; MRM conceived hypothesis, provided intellectual input, designed experiments, analyzed data, read and edited the paper. All authors read and approved the final manuscript.

## Supplementary Material

Additional file 1: Figure S1 Overview of gene expression microarray results. (A) Histogram showing distribution of log ratio of gene expression in 4 biological replicates of two cell lines (GN25-treated/untreated) measured by two-color Agilent assay. The histogram demonstrates that a greater number, and higher gene expression changes occurred in SNAIL overexpressing KRAS-HMLE-SNAIL cells than in KRAS-HMLE cells (HMLE-SNAIL, blue versus HMLE, red). (B) Comparison of numbers of gene transcripts showing significant change in each cell line due to treatment with GN25 (p≤.001; FC≥2; NCBI refseq and ENSEMBL transcripts). From the 2973 gene transcripts unique to the KRAS-HMLE-SNAIL context, 2737 were functionally annotated. These were used in subsequent systems-level analyses to investigate functional interaction networks and signaling pathways affected by GN-25 treatment. Click here for file

Additional file 2: Table S1 Predicted downstream functional effects due to GN-25 treatment of KRAS-HMLE-SNAIL cells. Analysis of significantly changed genes differentially affected by GN-25 in KRAS-HMLE-SNAIL cells compared to KRAS-HMLE cells. From an enrichment analysis with Ingenuity Systems^®^ software (Redwood, CA). Click here for file

Additional file 3: Table S2 Results from gene set comparisons in OncomineTM. The identified MYC-regulated genes significantly changed with GN-25 treatment in the KRAS-HMLE-SNAIL-specific data set (known myc-regulated genes in KRAS-HMLE-SNAIL) were used to survey of the Oncomine™ database. The results revealed that the MYC-targeted genes in the analyzed data set significantly overlapped with genes associated with an exceptionally poor prognosis in the database. Click here for file
