# Supplementary figures and images for: Systems analysis reveals a transcriptional reversal of the mesenchymal phenotype induced by SNAIL-inhibitor GN-25
Source: BMC Syst Biol. 2013 Sep 3;7:85. doi: 10.1186/1752-0509-7-85 (PMC3848843; doi:10.1186/1752-0509-7-85)

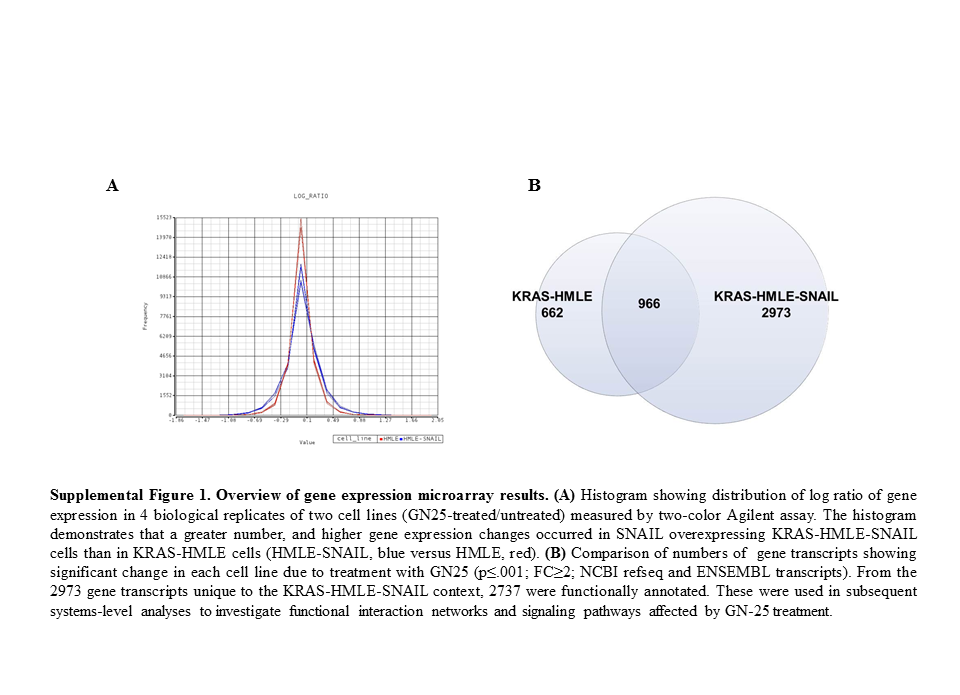

Supplement: Additional file 1: Figure S1 — Overview of gene expression microarray results. (A) Histogram showing distribution of log ratio of gene expression in 4 biological replicates of two cell lines (GN25-treated/untreated) measured by two-color Agilent assay. The histogram demonstrates that a greater number, and higher gene expression changes occurred in SNAIL overexpressing KRAS-HMLE-SNAIL cells than in KRAS-HMLE cells (HMLE-SNAIL, blue versus HMLE, red). (B) Comparison of numbers of gene transcripts showing significant change in each cell line due to treatment with GN25 (p≤.001; FC≥2; NCBI refseq and ENSEMBL transcripts). From the 2973 gene transcripts unique to the KRAS-HMLE-SNAIL context, 2737 were functionally annotated. These were used in subsequent systems-level analyses to investigate functional interaction networks and signaling pathways affected by GN-25 treatment. [file 1752-0509-7-85-S1.tiff]

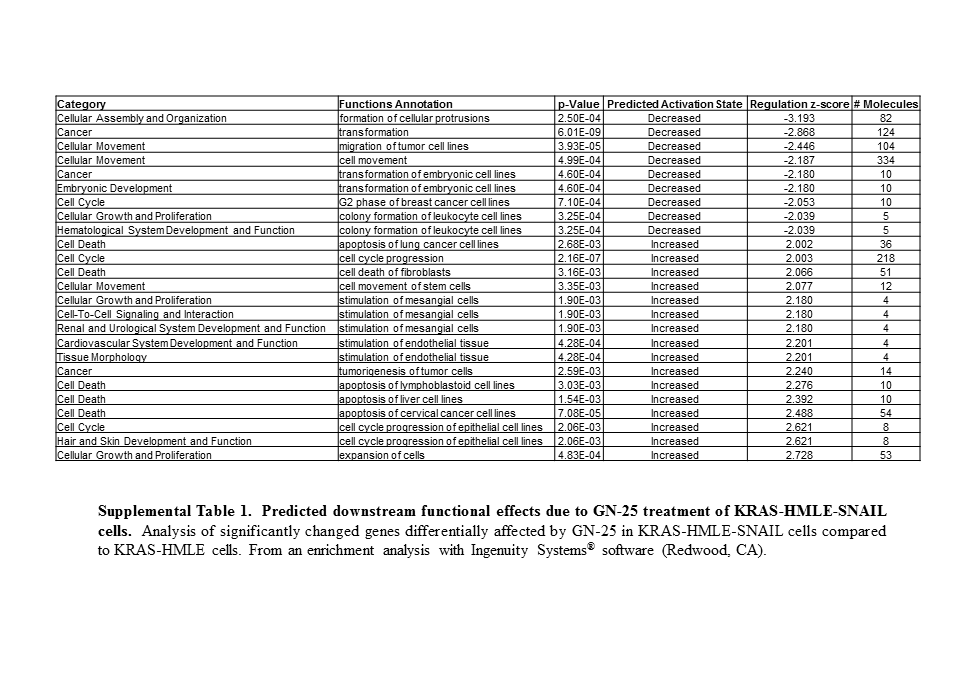

Supplement: Additional file 2: Table S1 — Predicted downstream functional effects due to GN-25 treatment of KRAS-HMLE-SNAIL cells. Analysis of significantly changed genes differentially affected by GN-25 in KRAS-HMLE-SNAIL cells compared to KRAS-HMLE cells. From an enrichment analysis with Ingenuity Systems® software (Redwood, CA). [file 1752-0509-7-85-S2.tiff]

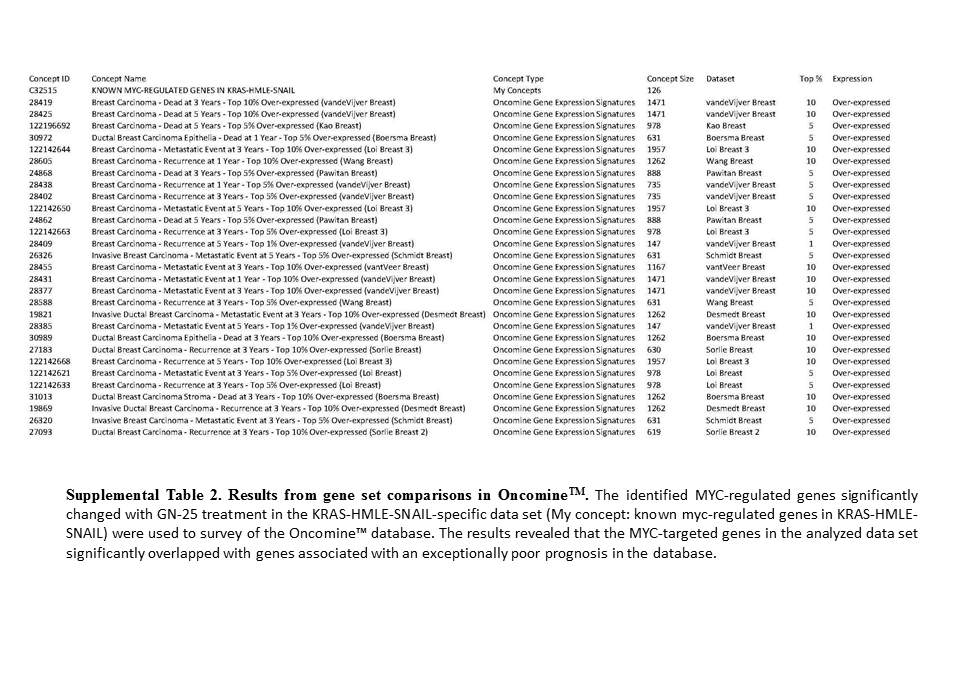

Supplement: Additional file 3: Table S2 — Results from gene set comparisons in OncomineTM. The identified MYC-regulated genes significantly changed with GN-25 treatment in the KRAS-HMLE-SNAIL-specific data set (known myc-regulated genes in KRAS-HMLE-SNAIL) were used to survey of the Oncomine™ database. The results revealed that the MYC-targeted genes in the analyzed data set significantly overlapped with genes associated with an exceptionally poor prognosis in the database. [file 1752-0509-7-85-S3.tiff]
